# Experiences, Lessons, and Challenges With Adapting REDCap for COVID-19 Laboratory Data Management in a Resource-Limited Country: Descriptive Study

**DOI:** 10.2196/50897

**Published:** 2024-04-16

**Authors:** Kagiso Ndlovu, Kabelo Leonard Mauco, Onalenna Makhura, Robin Hu, Nkwebi Peace Motlogelwa, Audrey Masizana, Emily Lo, Thongbotho Mphoyakgosi, Sikhulile Moyo

**Affiliations:** 1 Department of Computer Science University of Botswana Gaborone Botswana; 2 Department of Learning, Informatics, Management and Ethics Karolinska Institutet Stockholm Sweden; 3 College of Arts and Sciences University of Pennsylvania Philadelphia, PA United States; 4 National Health Laboratory Ministry of Health Gaborone Botswana; 5 Botswana Harvard AIDS Institute Partnership Gaborone Botswana

**Keywords:** REDCap, DHIS2, COVID-19, National Health Laboratory, eHealth, interoperability, data management, Botswana

## Abstract

**Background:**

The COVID-19 pandemic brought challenges requiring timely health data sharing to inform accurate decision-making at national levels. In Botswana, we adapted and integrated the Research Electronic Data Capture (REDCap) and the District Health Information System version 2 (DHIS2) platforms to support timely collection and reporting of COVID-19 cases. We focused on establishing an effective COVID-19 data flow at the national public health laboratory, being guided by the needs of health care professionals at the National Health Laboratory (NHL). This integration contributed to automated centralized reporting of COVID-19 results at the Ministry of Health (MOH).

**Objective:**

This paper reports the experiences, challenges, and lessons learned while designing, adapting, and implementing the REDCap and DHIS2 platforms to support COVID-19 data management at the NHL in Botswana.

**Methods:**

A participatory design approach was adopted to guide the design, customization, and implementation of the REDCap platform in support of COVID-19 data management at the NHL. Study participants included 29 NHL and 4 MOH personnel, and the study was conducted from March 2, 2020, to June 30, 2020. Participants’ requirements for an ideal COVID-19 data management system were established. NVivo 11 software supported thematic analysis of the challenges and resolutions identified during this study. These were categorized according to the 4 themes of infrastructure, capacity development, platform constraints, and interoperability.

**Results:**

Overall, REDCap supported the majority of perceived technical and nontechnical requirements for an ideal COVID-19 data management system at the NHL. Although some implementation challenges were identified, each had mitigation strategies such as procurement of mobile Internet routers, engagement of senior management to resolve conflicting policies, continuous REDCap training, and the development of a third-party web application to enhance REDCap’s capabilities. Lessons learned informed next steps and further refinement of the REDCap platform.

**Conclusions:**

Implementation of REDCap at the NHL to streamline COVID-19 data collection and integration with the DHIS2 platform was feasible despite the urgency of implementation during the pandemic. By implementing the REDCap platform at the NHL, we demonstrated the possibility of achieving a centralized reporting system of COVID-19 cases, hence enabling timely and informed decision-making at a national level. Challenges faced presented lessons learned to inform sustainable implementation of digital health innovations in Botswana and similar resource-limited countries.

## Introduction

The onset of the COVID-19 pandemic resulted in a global public health crisis [1]. The pandemic stretched almost all health care systems to their limits and exposed their weaknesses [2]. Previously documented COVID-19 challenges for the health sector include a lack of the health care services needed for the pandemic, inadequate resources, limited testing ability and capacity for a COVID-19 response, as well as overall poor data management within existing health care systems [1]. It was previously projected that countries in sub-Saharan Africa could see a sharp rise in COVID-19 infection rates and deaths, as such challenges are prominent in resource-limited countries [3]. This projected disproportionate impact of COVID-19 in resource-limited countries comes as no surprise. Currently, the health sector in high-income countries is deemed as underfunded, while in most resource-limited countries, it is reportedly heavily underfunded [4]. It is not a surprise that, in 2001, African leaders through the African Union’s Abuja Declaration agreed to “allocate 15% of the state’s annual budget to the improvement of the health sector” [5]. However, in 2013, only 5 African countries had achieved this target, while in 2018, only 2 countries achieved the target [6].

Access to accurate and current information has been recognized globally as a critical requirement for timely COVID-19 pandemic responses [7]. As such, the pandemic presented the need for robust data management systems in response to the evolving nature of COVID-19 [8]. Ideally, eHealth—“cost-effective and secure use of information and communications technologies (ICT) in support of health and health-related fields, including health-care services, health surveillance, health literature, and health education, knowledge and research” [9]—could ensure reliable information reporting and timely decision-making by governments and relevant stakeholders. However, requirements for such eHealth solutions present a greater challenge for resource-limited countries with documented weak ICT infrastructure, limited maintenance budgets, a lack of health human resource capacity to utilize eHealth systems, and nonuniform unique patient identifiers [10]. According to Archer et al [11], other factors affecting successful implementation of eHealth solutions in resource-limited countries include the absence of eHealth agendas, ethical and legal considerations, common system interoperability standards, and reliable power supplies.

Similar to other nations responding to the pandemic, the government of Botswana, through the Ministry of Health (MOH), identified eHealth as a means to improve COVID-19 data management and address complex data capture and transfer processes at various port of entries and the National Health Laboratory (NHL). Botswana’s eHealth infrastructure was previously reported as generally adequate, with functioning computers and some communication systems such as telephones, email, and Internet services [12]. According to Seitio-Kgokgwe et al [12], almost all public health facilities in Botswana are now connected to the government data network (GDN) with an average Internet connectivity of about 2 Mbps. The authors further highlighted that Botswana's eHealth initiatives have always operated within a very weak policy and regulatory framework characterized by inadequate health information legislation, national policy, and strategic plan. Fragmentation and inefficient eHealth initiatives were noted as other contributing factors to poor utilization of information in Botswana's health sector, as well as the lack of appreciation of the important role played by health information in managing health services. Currently, over 52 health laboratories operate in Botswana, 8 of which are accredited with national certifications like the South African National Accreditation System (SANAS), Clinical Laboratory Improvement Amendments (CLIA), International Organization for Standardization (ISO) 15189, or the Southern African Development Community Accreditation Services (SADCAS) [13,14].

The Botswana NHL is one of the accredited laboratories tasked with the management of national COVID-19 testing. The NHL has decentralized its COVID-19 testing services from the capital city Gaborone, by operating satellite testing centers in selected districts with sizable populations and key ports of entry into the country. The NHL data flow is such that specimen data go through each of the 4 laboratory stages of (1) Reception Lab (all incoming lab specimens are captured using a barcode scanner), (2) Extraction Lab (specimen processing for nucleic acid isolation and purification), (3) Detection Lab (sample amplification and detection), and (4) Resulting and Verification Lab (lab results are captured and verified for release to clients). The need to scan laboratory specimen samples at each of the laboratory phases is an important quality assurance step. Accession numbers in laboratory processes are critical to link the specimen with a participant. Despite having an already existing eHealth system at the NHL, electronic data transfers within and across the 4 laboratories were considered tedious, time consuming, and a risk to both data quality and timely COVID-19 results reporting. These limitations required immediate attention.

The authors volunteered their technical support toward implementation of a customizable COVID-19 data management system—Research Electronic Data Capture (REDCap)—at the NHL. REDCap was suggested by the authors following its documented benefits including its utility within a resource-limited country context [15,16], as well as its availability locally through the University of Botswana (UB). REDCap is a secure, web-based platform designed to support electronic data capture. It was developed at Vanderbilt University in the United States in 2004 and can be set up to support a variety of health care environments and scenarios. REDCap is compliant with international standards such as the Health Insurance Portability and Accountability Act (HIPAA) and General Data Protection Regulation (GDPR).

In order to support timely reporting of COVID-19 cases, the REDCap platform was linked to the District Health Information System version 2 (DHIS2) at the MOH. DHIS2 is another open-source platform for collecting, processing, and analyzing health data. It was developed and first implemented in 1998 by the Health Information System Programme (HISP) in South Africa and offers a secure web-based electronic data capture, analysis, and reporting tool [17]. Similar to REDCap, the DHIS2 platform has a mobile application (DHIS Tracker), enabling its use in case of weak and fluctuating Internet connectivity. The DHIS2 Tracker allows for auto-syncing of data with the central server once stable Internet connectivity returns. The data elements shared between DHIS2 and REDCap include COVID-19 test results and demographic information of those who were tested including their names, gender, ages, locations, and related underlying medical conditions.

Through a participatory design approach [18,19], the REDCap platform was customized to support the NHL data process needs, with the goal of increasing operational efficiency and reducing overreliance on paper-based manual approaches. The authors’ preference for a participatory design approach aligns with that of Berntsen [20] who acknowledged that initial involvement of end users is central to the development of a health information system. This study reports on the experiences, challenges, and lessons learned while designing, adapting, and implementing REDCap and the DHIS2 platform to support COVID-19 data management processes at the NHL in Botswana.

## Methods

A participatory design approach was adopted to guide the design, customization, and implementation of REDCap for COVID-19 data management at the NHL.

### Study Population, Setting, and Design

#### Participants

All NHL and MOH personnel responsible for processing COVID-19 specimen samples and data were invited to participate. All potential participants were sent an introductory email describing the background and objectives of the study. A consent form was subsequently shared with all those who showed interest. All invited participants agreed to participate in the study, of which 29 were NHL personnel and 4 were based at the MOH. Of the 29 NHL personnel, 12 were based at the Reception Lab, and 17 were based at the Extraction, Detection, and Resulting and Verification Labs. The study spanned from March 2, 2020, to June 30, 2020.

Data collection was conducted in 3 phases.

#### Phase 1: Participant Engagement

The authors facilitated a 1-day consultative physical meeting or workshop with the study participants to solicit specific requirements for the COVID-19 data management system. Study participants were requested to define requirements for an ideal COVID-19 data management system. The authors recorded all participants' responses during the session which lasted for 1 hour. Based on the insights gathered from this exercise, iterative design and testing approaches were adopted for each key deliverable from design, customization, and implementation of the REDCap platform to support COVID-19 data management. A minimum of 2 iterations and a maximum of 4 iterations were incurred per deliverable, and this was influenced by the complexity or noncomplexity of the tasks.

#### Phase 2: Implementation and Assessment of the Platform to Meet User Requirements

Design and customization of the REDCap platform were followed by implementation of the solution as well as evaluating its feasibility to address the previously noted requirements and specifications. This involved participants testing the REDCap platform and providing feedback on any issues or challenges they encountered.

#### Phase 3: Human Resource Capacity Development

At each phase of the design, customization, and implementation of the REDCap platform, participants were trained on the various system components and had the opportunity to share their feedback to inform next steps.

### Ethical Considerations

This study was approved by the Ethics Committee of the University of Botswana (Reference: UBR/RES/IRB/BIO/GRAD/244). All data experiments were performed in accordance with relevant guidelines and regulations such as the Declaration of Helsinki. All participants were informed of the objective of the exercise as well as their voluntary participation, and all gave informed consent for this study. Those who consented to participate were immediately sensitized and granted access to the REDCap instance at the UB. No compensation was provided for participating in the study.

### Data Analysis

NVivo 11 software was used for thematic analysis [21] of data to determine participants’ requirements for an ideal COVID-19 data management system and their postimplementation experiences.

## Results

Study participants consisted of all personnel involved with handling COVID-19 specimens and managing all relevant laboratory data at both the NHL and MOH (Table 1).

**Table 1:** Study participants and their roles in COVID-19 data management.

Participant type	Participants, n	Roles in the study
National Health Laboratory (NHL) Reception Lab	12	Scanning of COVID-19 specimen samples at point of reception
NHL Extraction Lab	5	Scanning of COVID-19 specimen samples to create maps/batch lists and for nucleic acid extraction
NHL Detection Lab	7	Scanning of COVID-19 specimen samples into the analyzers for the detection of SARS-CoV-2
NHL Resulting and Verification Lab	5	Resulting and verification of COVID-19 specimen results for access in Research Electronic Data Capture (REDCap) and producing lab reports
Health informatics personnel	4	Served as intermediaries between clinical lab personnel and IT personnel

Consultative meetings with study participants led to the identification of user requirements for an ideal COVID-19 data management system at the NHL. These were categorized under the following 2 themes: functional and nonfunctional requirements (Table 2).

**Table 2:** Thematic presentation of participants’ requirements for the COVID-19 data management system.

Requirement type	Requirements
Functional requirements	“Authentication by unique username and password” “Automated sign out in case of user inactivity” “Data validation checks” “Temporary data staging between the two systems (REDCap^a^ and DHIS2^b^)” “Automated field data pull from the DHIS2 system” “Automated COVID-19 results sync with the DHIS2 system” “Data summary dashboard” “Barcode data export for data capture, resulting, and verification” “Automated data backups” “Data encryption during transfer and storage” “Automated alert message in case of a positive COVID-19 result” “Reordering of barcodes to be displayed on the detection plate to resemble the 8x12 matrix and preferably printed on two pages”
Nonfunctional requirements	“High performance system” “Reliable data management system” “User-friendly system” “Scalable” “Cross-platform independent” “Secure system” “Interoperable system”

^a^REDCap: Research Electronic Data Capture.

^b^DHIS2: District Health Information System version 2.

**Figure 1:**
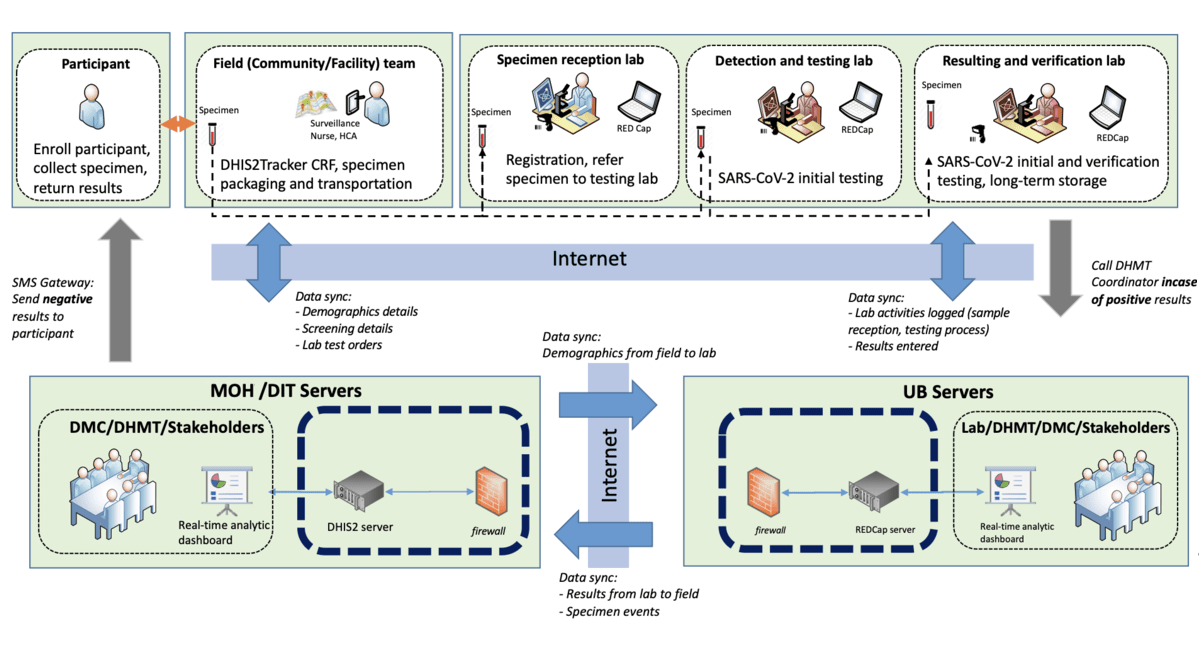
Data flow architecture design for COVID-19 data management at the National Health Laboratory (NHL). CRF: case report form; DHIS2: District Health Information System version 2; DHMT: District Health Management Team; DIT: Department of Information Technology; DMC: Data Management Committee; MOH: Ministry of Health; REDCap: Research Electronic Data Capture; UB: University of Botswana.

**Figure 2:**
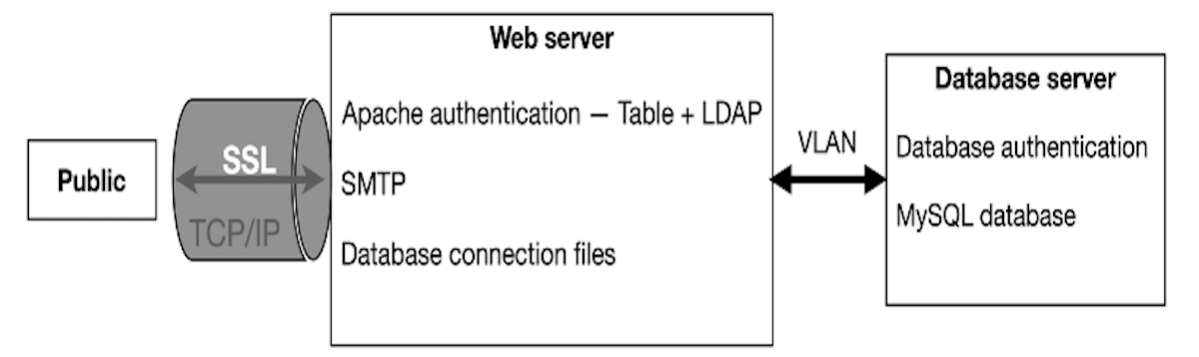
Research Electronic Data Capture (REDCap) security architecture supporting COVID-19 data management at the National Health Laboratory (NHL). LDAP: Lightweight Directory Access Protocol; SMTP: Simple Mail Transfer Protocol; SSL: Secure Sockets Layer; VLAN: virtual local area network.

**Figure 3:**
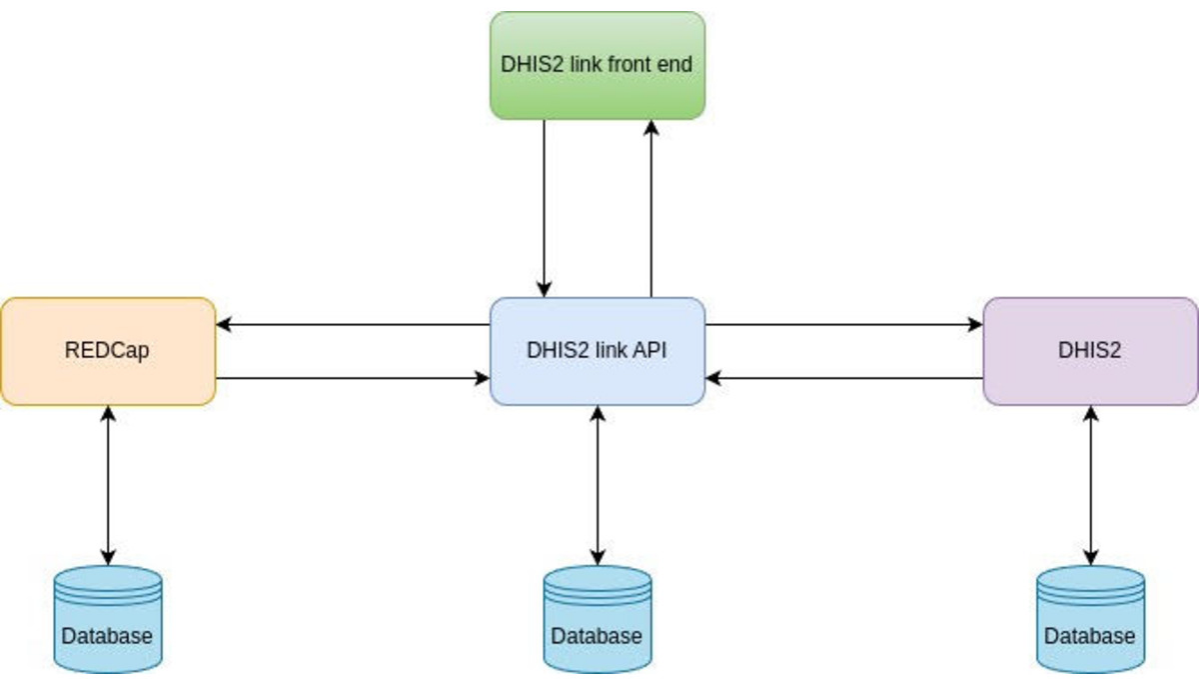
High-level view of the link between the District Health Information System version 2 (DHIS2) and Research Electronic Data Capture (REDCap). API: application programming interface.

**Figure 4:**
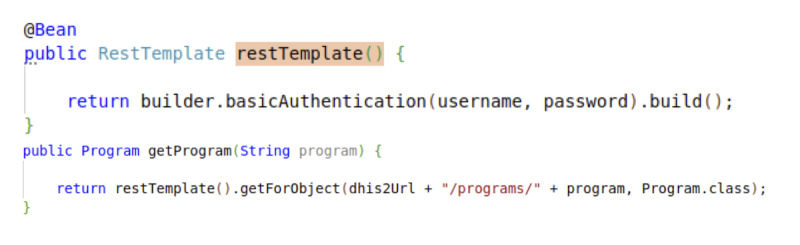
Sample code for using the RestTemplate object to access the programs configured in District Health Information System version 2 (DHIS2).

**Figure 5:**
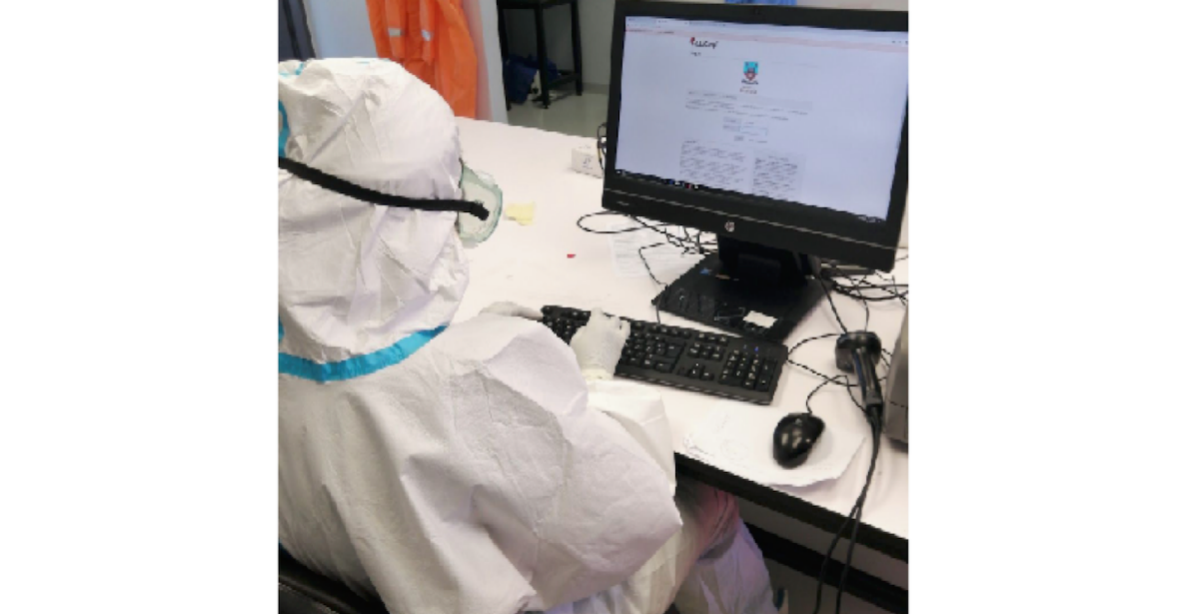
Specimen Receiving Lab personnel capturing data using Research Electronic Data Capture (REDCap).

**Figure 6:**
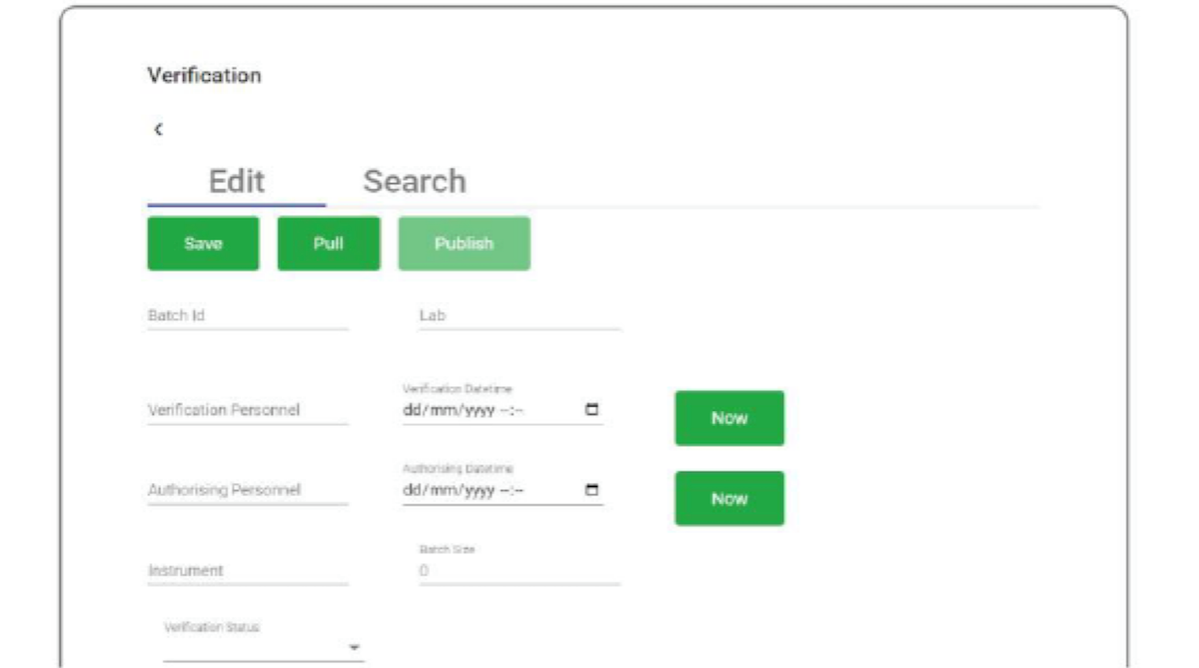
Angular web application for pulling records from District Health Information System version 2 (DHIS2) and storage in Research Electronic Data Capture (REDCap).

**Figure 7:**
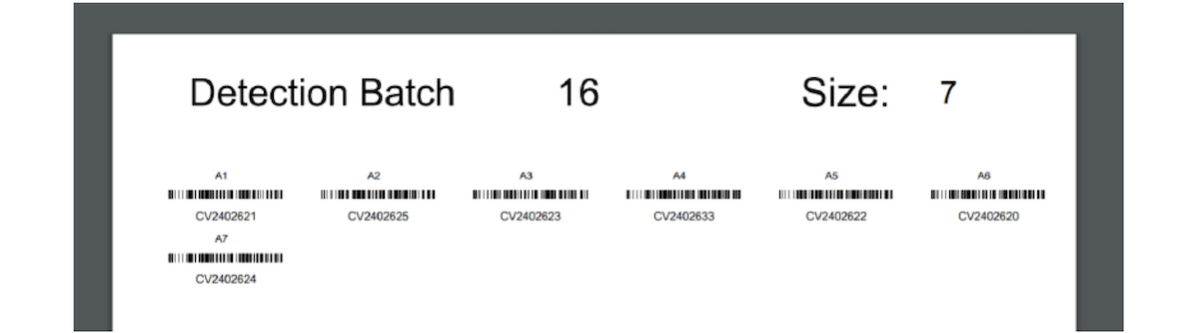
Sample barcodes generated from the Angular web application.

Although this paper reports on challenges and resolutions while implementing REDCap at the NHL (Table 3), Figures 1-7 are intended to illustrate the design, customization, and implementation of the REDCap platform at the NHL.

**Table 3:** Challenges and resolutions while implementing Research Electronic Data Capture (REDCap) at the National Health Laboratory (NHL).

Challenges	Resolutions
Network firewall constraints to access REDCap within the government data network (GDN)	Ministry of Health (MOH) leadership engaged relevant departments to resolve firewall constraints.
Misalignment of barcode scanner configuration	The barcode scanner was reconfigured to allow acceptance of the “tab” key versus “carriage return” key upon capturing a specimen. Computers were set up specifically for REDCap use. 2 scanners were used: one dedicated for REDCap use and another for the pre-existing eHealth system.
Lack of trained NHL personnel	REDCap users were continuously sensitized and trained. User manuals and training slides were developed and shared with the rest of the implementation team.
Slow Internet connection speed at the NHL (download and upload speeds of 12.59 Mbps and 16.84 Mbps, respectively)	Relevant government departments were informed of the slow internet connectivity, and attempts were made to improve the Internet bandwidth. Mobile WiFi routers from private Internet service providers (ISPs) were used to assist during the training sessions.
Failure of REDCap to automatically pull data from the District Health Information Software version 2 (DHIS2) platform	A web application programming interface (API) was developed by the authors and supported pulling data from the DHIS2 system, subsequently imported into the REDCap platform.
REDCap unable to generate the barcodes, posing a big issue at the extraction lab	A web portal/application was developed by the authors and linked to the REDCap platform. The application supported generating unique barcodes for each of the COVID-19 specimens.
Reordering of barcodes to be displayed on the detection plate to resemble the 8x12 matrix and getting those printed on 2 pages	Although not a 2-page 8x12 matrix, the third-party web application developed by the authors supported the ordering of barcodes and reformatting them into a printer-friendly format.
Need for a comment field in the REDCap result form	The authors added a comment field on the REDCap result form.

The authors used participants’ feedback to guide the design of an ideal COVID-19 data flow architecture for this study (Figure 1).

The REDCap data security architecture at UB separates the web server from the database server (Figure 2).

In order to facilitate timely decision-making and address a key technical requirement (“Automated COVID-19 results sync with the DHIS2 system” in Table 2), the REDCap system was linked with the DHIS2 system at the MOH for aggregate data reporting.

Interfacing between DHIS2 and REDCap was controlled by the DHIS2 link application programming interface (API; Figure 3). The DHIS2 API link supported 3 functionalities: (1) data format conversions between DHIS2 and REDCap, (2) avoiding duplicate synchronizing of data between REDCap and DHIS2, and (3) enabling data access for the DHIS2 front end, which relied on the API to make consistent data available for the 2 systems.

In order to access the DHIS2 API, a RestTemplate object short code was implemented with basic authentication secured by username and password (Figure 4).

Study participants accessed REDCap using their personal account details (unique usernames and passwords) before proceeding to collect, manage, and report on COVID-19 at the MOH (Figure 5).

Of important note, the REDCap platform triggered automated email alerts whenever a COVID-19 positive result was recorded. The email alerts were sent to study coordinators at the MOH.

A third-party web application simplified the process of pulling records from the DHIS2 system into REDCap (Figure 6) and enabled the viewing of specimen barcodes through a web browser (Figure 7).

Challenges and resolutions while implementing the REDCap platform to support COVID-19 data management at the NHL are summarized in Table 3.

## Discussion

This paper describes the experiences, challenges, and lessons learned while designing, customizing, and implementing the REDCap platform for COVID-19 data management at the NHL in Botswana. REDCap implementation challenges and lessons identified during the study were categorized under the following 4 themes: (1) infrastructure, (2) capacity development, (3) platform constraints, and (4) interoperability.

### Infrastructure

The literature emphasizes the critical need for robust ICT infrastructure to serve as the backbone for successful and sustainable digital health implementation [22-25]. The national eHealth strategy tool kit by the World Health Organization (WHO) and International Telecommunication Union (ITU) identifies essential eHealth infrastructure components as high-speed data connectivity, computing infrastructure, identification and authentication services, directory services, health care provider systems, electronic health record repositories, and health information data sets, all of which underpin a national eHealth environment [26]. The tool kit further urges countries to secure long-term funding for investment in national eHealth infrastructure and services. It is against this backdrop that the WHO recognizes a pressing need for countries to invest in infrastructure to support digital transformation; Internet connectivity; and issues related to legacy infrastructure, technology ownership, privacy, and security while adapting and implementing globally recognized standards and technologies [27].

In this study, infrastructural challenges experienced include weak Internet speed and misalignment of computer network firewalls between the GDN and the UB network. These issues resonate with the identified ICT infrastructural challenges within the Botswana National eHealth strategy [28]. During implementation of the REDCap platform at the NHL, initial Internet download and upload speeds were 12.59 Mbps and 16.84 Mbps, respectively (Table 3). The Federal Communications Commission (FCC) considers minimum Internet speeds of 5 Mbps to 25 Mbps as ideal to support online tasks such as file download and telecommuting [29]. However, despite meeting the FCC requirement, study participants reported the need for the Internet speed to be upgraded to adequately support frequent data transactions and data sharing between REDCap and the DHIS2 platform. This could be due to multiple factors. The following are some example quotes from study participants pertaining to their dissatisfaction with the Internet bandwidth:

Internet connectivity is still slow.

participant, NHL Reception Lab

Internet bandwidth needs to be upgraded.

participant, NHL Resulting and Verification Lab

As a mitigation for the slow Internet connectivity, relevant government departments were engaged, and measures were put in place to increase the Internet speed. This includes procurement of mobile Internet routers from private internet service providers (ISPs) for use during the study. The engagement of private sector stakeholders to support implementation of eHealth initiatives is also highlighted as essential within the “Strategy and Investment” pillar of the Botswana National eHealth Strategy, which emphasizes “eHealth planning, with involvement of major stakeholders and sectors” [28].

Another challenge was the restriction introduced by computer network firewalls resulting in data flow constraints between the GDN and UB networks. A similar issue was encountered in another study, in which implementation of the REDCap platform incurred network firewall challenges hindering participants from using computers outside the Veterans Health Administration network to complete a survey on REDCap [30]. According to Nagpure et al [31], firewall policy conflicts can be complex to eliminate but could be addressed through practical resolution methods such as the “first-match resolution” involving “identifying which firewall policy rule involved in a conflict situation should take precedence when multiple conflicting rules (with different actions) filter a particular network packet simultaneously.” At the NHL, the authors resolved the network firewall challenges by engaging relevant ICT authorities at both governmental and UB IT departments to facilitate reconfiguration of the firewall settings to allow data flow between the 2 networks. This necessitated effective measures for data security, privacy, and confidentiality throughout the study. To achieve this, the authors ensured that the REDcap and DHIS2 servers communicate through an encrypted Internet connection using a 128-bit Secure Sockets Layer (SSL) certificate. The use of SSL has been previously considered a standard technology for securing electronic commerce and electronic banking transactions over the Internet [32]. However, recent advances in cybersecurity attacks call for using technologies to detect compromised SSL network traffic [33]. This consideration was brought to the attention of both governmental and UB IT departments. Compliance with the Botswana-specific Data Protection Act [34] could improve the necessary safeguards for the right to privacy of individuals and the collection and transfer of their personal data.

### Capacity Development

Some study participants lacked the requisite skills to effectively use the REDCap platform, which was another challenge encountered during the study. The lack of competent and experienced REDCap users in some low and middle-income countries (LMICs) has been linked to poor “REDCap penetration” in those countries [35]. For example, in Botswana, only 3 institutions have REDCap instances [36], and unless affiliated with any of these institutions, most health care workers are unfamiliar with the platform. Low ICT literacy is common among health care workers in most LMICs [25] and could affect the ability of some participants to competently use the REDCap platform. Recognizing this challenge, the Botswana National eHealth strategy included “Workforce Development” as one of its pillars dedicated to eHealth capacity building among health care workers [28].

A reported benefit of having a health workforce trained in ICT prior to engaging with a health information system is minimizing avoidable errors, which, in this case, may be influenced by a lack of familiarity with the REDCap platform or poor ICT competency. To ensure the effective use of REDCap at the NHL, the authors continuously trained participants at all stages in the study. The ever-evolving nature of the REDCap platform [16,36] also necessitates that those working with the system must continuously familiarize themselves with its core functionalities and data workflows through support from the REDCap Consortium. Almost all training needs for this study were identified during weekly virtual progress report meetings with all study participants. Training content was also informed by the identified system requirements for the REDCap platform (Table 2). According to Nsaghurwe et al [37], “functional requirements describe what the system should do—such as its ability to exchange client-level data in a single repository, search for records with data quality issues; while non-functional requirements describe how the system should perform,” including system performance, reliability, and user-friendliness.

In essence, a central aspect to the successful implementation of any health information management system is the need to train users on how to appropriately utilize the system to capture and manage data [25]. Recommended approaches from the existing literature include ensuring continuous engagement and availing training materials for trainees' access and reference at a later stage. In this study, the authors made all training material and manuals available via the REDCap “File Repository” feature for the participants to access at their convenience posttraining.

Informed by the level of participants’ familiarity with the REDCap platform, other measures were put in place such as denying participants “data deletion” privileges on the REDCap system. Instead, participants could add, edit, and view data they required to complete their respective tasks. This minimized accidental data deletion and resonated with the famous “principle of least privilege” by Saltzer and Schroeder [38], which states that “every program and every user of the system should operate using the least set of privileges necessary to complete the job.”

### Platform Constraints

An important lesson learned was that, although REDCap is often regarded as a complete solution for data management [16], in some instances, support from other software applications could augment its limitations. In this study, the REDCap platform was not able to generate barcodes for the NHL personnel to scan to retrieve previously captured information. This limitation was resolved by developing a web-based application supported by the Angular framework [39] for quick production of scannable barcodes required during the Results and Verification Lab stage at the NHL. Some documented benefits of the Angular framework include support for lightweight web applications; faster software development capabilities; and easily readable, testable, and interoperable software solutions [39]. For this study, the authors leveraged the ability to easily link the Angular web application with the REDCap platform. Moreover, only one username and password combination was used to access the data capture forms between the Angular-based web application and REDCap.

Another constraint encountered was the inability of the REDCap platform to automatically store COVID-19 specimen barcodes on the data collection form. Further, the REDCap system requires that the barcode scanner accept a “tab key” to move to the next field, but the scanner configuration used at the NHL accepted a “carriage return key” instead to move to the next field. This meant that each time a barcode was scanned by the study participants, REDCap would not capture it into the appropriate field unless the user pressed the “tab key” on the keyboard to trigger a move to the next field. This became tedious to participants considering the high number of barcodes requiring scanning at the NHL during the COVID-19 pandemic. To resolve this, the authors reconfigured the barcode scanners to align with REDCap requirements, that is, use of the “tab key” to capture barcodes versus the initial configuration of the “carriage return key.” Moreover, the MOH procured dedicated barcode scanners for use with the REDCap platform as the previous ones accompanied a different system at the NHL. Overall, REDCap ensured auto-saving of the barcodes as they are being scanned, minimizing data loss in case of network or power failure.

### Interoperability

The Healthcare Information and Management Systems Society (HIMSS) defines interoperability as “the ability of different information systems, devices, and applications (‘systems’) to access, exchange, integrate and cooperatively use data in a coordinated manner, within and across organizational, regional and national boundaries, to provide timely and seamless portability of information and optimize the health of individuals and populations globally” [40]. Some documented benefits of interoperable health care information systems include improved patient management, quality of care, and decision-making, as well as reduced health care costs [41]. However, several challenges have been noted that hinder the interoperability of health care systems, affecting their successful and sustainable implementation, especially in resource-limited countries [10].

Despite the documented interoperability challenges, REDCap has been successfully linked to other systems previously. For example, a study in the United Kingdom automated the process by which COVID-19 clinical trial registration records were exported from the WHO International Clinical Trials Registry Platform into external software [42]. A linking script subsequently pulled relevant data, aligned it with the data dictionary in REDCap, and directly imported it, removing any duplicates in the process. Other researchers utilized REDCap as a data harmonizer, managing and combining cancer research data from multiple registries and allowing for the reconciliation of disparate data sets as well as their conversion [43,44]. In another previous study, REDCap served as the platform housing all global data collected with modified survey questions to capture nuances and allowed for individualization to study public health interventions for COVID-19 [45]. Consequently, these applications reinforced REDCap’s utility for cross-continental collaborations, extending beyond any singular institution or setting.

In this study, although linking the REDCap platform with the DHIS2 platform was achieved, the failure of the REDCap system to automatically pull data from the DHIS2 platform was noteworthy. This limitation was a result of constraints within the Dynamic Data Pull (DDP) feature in REDCap. The REDCap DDP is a special feature for importing data into REDCap from an external source system [46]. The DDP feature provides an adjudication process whereby REDCap users can approve all incoming data from the source system before the data are officially saved in their REDCap project. Because the REDCap DDP assumes that all incoming data from the source system may not be trusted as valid or that only a subset of the data coming from the source system needs to be imported, it utilizes an adjudication web page inside REDCap’s interface to allow manual review of the data obtained from the source system before confirming that it be imported into the REDCap repository [46]. It is precisely the adjudication process that hindered the automatic data import from the DHIS2 system into REDCap. To address this challenge, a custom API was developed by the authors to support accessing health records from the DHIS2 platform, staging them for quality assurance as a batch and automatically loading them into the REDCap system. Further, this approach helped minimize network traffic whenever data synchronization between REDCap and the DHIS2 systems occurred.

### Limitations

The REDCap system was implemented at the NHL in response to the emergency COVID-19 pandemic. Consequently, a systematic approach to its implementation could have been compromised, and essential considerations could have been overlooked. Most notably, multiple training and feedback sessions had to be conducted virtually using the Zoom platform due to restrictions on in-person gatherings. Hence, the authors were unable to frequently visit the NHL in person to provide the necessary technical support and training. Participation in the study was limited and often disrupted, as some NHL personnel were reassigned to efforts such as vaccine distribution or other scenarios not applicable for REDCap use. Last, increased workloads for participants due to COVID-19 surges in Botswana likely contributed to the lack of thorough quality checks when interacting with the system.

### Conclusion

Implementation of the REDCap platform to support COVID-19 data management at the NHL in Botswana was successful, albeit with challenges. It is worth noting that, like any software, REDCap as a system possesses limitations that were addressed with other applications to meet requirements for this study. Most challenges encountered were exacerbated by the lack of pandemic response preparedness, as was the case in Botswana and around the world. As such, most of these challenges will not be unique to this specific case of REDCap implementation but will continue to affect the sustainable implementation of eHealth innovations until conscious efforts using key digital health strategies are made that create an enabling environment to support implementation of digital health innovations. Another lesson learned is the essential need for collaborations with key stakeholders to minimize technological barriers while implementing eHealth solutions. Despite the challenges encountered, the REDCap and DHIS2 platforms served as readily available and customizable platforms to address COVID-19 data management at the NHL. To this end, effective planning is essential, including the engagement and training of key personnel to optimize the use of eHealth systems beyond the COVID-19 pandemic.

## Abbreviations

API: application programming interface
CLIA: Clinical Laboratory Improvement Amendments
DDP: Dynamic Data Pull
DHIS2: District Health Information System version 2
FCC: Federal Communications Commission
GDN: government data network
GDPR: General Data Protection Regulation
HIMSS: Healthcare Information and Management Systems Society
HIPAA: Health Insurance Portability and Accountability Act
HISP: Health Information System Programme
ICT: information and communication technology
ISO: International Organization for Standardization
ISP: private internet service provider
ITU: International Telecommunication Union
LMICs: low and middle-income countries
MOH: Ministry of Health
NHL: National Health Laboratory
REDCap: Research Electronic Data Capture
SADCAS: Southern African Development Community Accreditation Services
SANAS: South African National Accreditation System
SSA: sub-Saharan Africa
SSL: Secure Sockets Layer
UB: University of Botswana
WHO: World Health Organization

## Data Availability

The datasets used and/or analyzed during the study are available from the corresponding author on reasonable request.

## References

[1] Tessema GA, Kinfu Y, Dachew BA, Tesema AG, Assefa Y, Alene KA, Aregay AF, Ayalew MB, Bezabhe WM, Bali AG, Dadi AF, Duko B, Erku D, Gebrekidan K, Gebremariam KT, Gebremichael LG, Gebreyohannes EA, Gelaw YA, Gesesew HA, Kibret GD, Leshargie CT, Meazew MW, Mekonnen A, Mirkuzie AH, Mohammed H, Tekle DY, Tesfay FH (2021). The COVID-19 pandemic and healthcare systems in Africa: a scoping review of preparedness, impact and response. BMJ Glob Health.

[2] Lal A, Erondu NA, Heymann DL, Gitahi G, Yates R (2021). Fragmented health systems in COVID-19: rectifying the misalignment between global health security and universal health coverage. The Lancet.

[3] Adams J, MacKenzie MJ, Amegah AK, Ezeh A, Gadanya MA, Omigbodun A, Sarki AM, Thistle P, Ziraba AK, Stranges S, Silverman M (2021). The conundrum of low COVID-19 mortality burden in sub-Saharan Africa: myth or reality?. Glob Health Sci Pract.

[4] (2016). Global diffusion of eHealth: making universal health coverage achievable: report of the third global survey on eHealth. World Health Organization.

[5] Piabuo SM, Tieguhong JC (2017). Health expenditure and economic growth - a review of the literature and an analysis between the economic community for central African states (CEMAC) and selected African countries. Health Econ Rev.

[6] Gatome-Munyua A, Olalere N (2020). Public financing for health in Africa: 15% of an elephant is not 15% of a chicken. African Renewal.

[7] Field E, Dyda A, Lau CL (2021). COVID-19 Real-time Information System for Preparedness and Epidemic Response (CRISPER). Med J Aust.

[8] Lee SM, Lee D (2020). Lessons learned from battling COVID-19: the Korean experience. Int J Environ Res Public Health.

[9] eHealth. World Health Organization.

[10] Ndlovu K, Scott RE, Mars M (2021). Interoperability opportunities and challenges in linking mhealth applications and eRecord systems: Botswana as an exemplar. BMC Med Inform Decis Mak.

[11] Archer N, Lokker C, Ghasemaghaei M, DiLiberto D (2021). eHealth implementation issues in low-resource countries: model, survey, and analysis of user experience. J Med Internet Res.

[12] Seitio-Kgokgwe O, Gauld RD, Hill PC, Barnett P (2015). Development of the National Health Information Systems in Botswana: pitfalls, prospects and lessons. Online J Public Health Inform.

[13] Gaborone: Southern Africa Development Community Accreditation Services. Southern African Development Community Accredited Laboratories.

[14] Mokobela K Has Botswana reached a "tipping point" in lab accreditation. Strengthening Laboratory Management Toward Accreditation.

[15] Gadsden T, Bateman-Steel CR, Chaverot S, Ressler K, Chee K, Redwood L, Ferson MJ (2021). Using a computerised database (REDCap) to monitor influenza vaccination coverage of healthcare workers and staff in South Eastern Sydney Local Health District. Aust. Health Review.

[16] Harris PA, Taylor R, Minor BL, Elliott V, Fernandez M, O'Neal L, McLeod L, Delacqua G, Delacqua F, Kirby J, Duda SN, REDCap Consortium (2019). The REDCap consortium: Building an international community of software platform partners. J Biomed Inform.

[17] Braa JA, Sahay S, Celi LAG, Fraser HSF, Nikore V, Osorio JS, Paik K (2017). The DHIS2 Open Source Software Platform: Evolution Over Time and Space. Global Health Informatics.

[18] Schuler D, Namioka A (1993). Participatory Design: Principles and Practices.

[19] Ozkaynak M, Sircar CM, Frye O, Valdez RS (2021). A systematic review of design workshops for health information technologies. Informatics.

[20] Berntsen SN (2015). Enabling Participatory Design in low resource contexts. University of Oslo.

[21] Terry G, Hayfield N, Clarke V, Braun V, Willig C, Rogers WS (2017). Thematic Analysis. The SAGE Handbook of Qualitative Research in Psychology.

[22] Labrique AB, Wadhwani C, Williams KA, Lamptey P, Hesp C, Luk R, Aerts A (2018). Best practices in scaling digital health in low and middle income countries. Global Health.

[23] Anwar F, Shamim A (2011). Barriers in Adoption of Health Information Technology in Developing Societies. International Journal of Advanced Computer Science and Applications.

[24] Wamoto FO (2015). E-government Implementation in Kenya, an evaluation of Factors hindering or promoting e-government successful implementation. International Journal of Computer Applications Technology and Research.

[25] Kaboré SS, Ngangue P, Soubeiga D, Barro A, Pilabré AH, Bationo N, Pafadnam Y, Drabo KM, Hien H, Savadogo GBL (2022). Barriers and facilitators for the sustainability of digital health interventions in low and middle-income countries: A systematic review. Front Digit Health.

[26] (2012). National eHealth Strategy Toolkit. World Health Organization.

[27] (2021). Global strategy on digital health 2020-2025. World Health Organization.

[28] (2020). The eHealth strategy of Botswana (2020-2024). University of Botswana.

[29] Broadband Speed Guide. Federal Communications Commission.

[30] Paris B, Hynes D (2019). Diffusion, implementation, and use of Research Electronic Data Capture (REDCap) in the Veterans Health Administration (VA). JAMIA Open.

[31] Nagpure R, Dhuri PJ, Patil JK, Kini MP, Patil AJ (2015). Detection and resolution of firewall policy anomalies. The International Journal of Science and Technoledge.

[32] Raghavan K, Desai MS, Rajkumar PV (2017). Managing cybersecurity and ecommerce risks in small businesses. Journal of Management Science and Business Intelligence.

[33] Ghafir I, Prenosil V, Hammoudeh M, Han L, Raza U (2017). Malicious SSL Certificate Detection: A Step Towards Advanced Persistent Threat Defence.

[34] Data Protection Act, 2018: Law 32. Botswana Communications Regulatory Authority.

[35] Van Bulck L, Wampers M, Moons P (2022). Research Electronic Data Capture (REDCap): tackling data collection, management, storage, and privacy challenges. Eur J Cardiovasc Nurs.

[36] Harris PA, Taylor R, Thielke R, Payne J, Gonzalez N, Conde JG (2009). Research electronic data capture (REDCap)--a metadata-driven methodology and workflow process for providing translational research informatics support. J Biomed Inform.

[37] Nsaghurwe A, Dwivedi V, Ndesanjo W, Bamsi H, Busiga M, Nyella E, Massawe JV, Smith D, Onyejekwe K, Metzger J, Taylor P (2021). One country's journey to interoperability: Tanzania's experience developing and implementing a national health information exchange. BMC Med Inform Decis Mak.

[38] Saltzer J, Schroeder M (1975). The protection of information in computer systems. Proc. IEEE.

[39] Angular.

[40] Interoperability in Healthcare. Healthcare Information and Management Systems Society.

[41] Olaronke I, Soriyan A, Gambo I, Olaleke J (2013). Interoperability in healthcare: benefits, challenges and resolutions. International Journal of Innovation and Applied Studies.

[42] Maguire BJ, Guérin PJ (2020). A living systematic review protocol for COVID-19 clinical trial registrations. Wellcome Open Res.

[43] Betti M, Bertolotti M, Roveta A, Bolgeo T, Cassinari A, Delorenzi S, Dacquino MT, Giacchero F, Pelazza C, Penpa S, Viazzi F, Zichitella D, Maconi A (2020). Creation of a database through the REDCap web-interface for the data collection of COVID-19 patients admitted to the Alessandria Hospital. Working Paper of Public Health.

[44] Whisenant JG, Trama A, Torri V, De Toma A, Viscardi G, Cortellini A, Michielin O, Barlesi F, Dingemans AC, Van Meerbeeck J, Pancaldi V, Soo RA, Leighl NB, Peters S, Wakelee H, Garassino MC, Horn L (2020). TERAVOLT: Thoracic Cancers International COVID-19 collaboration. Cancer Cell.

[45] Zheng Q, Jones FK, Leavitt SV, Ung L, Labrique AB, Peters DH, Lee EC, Azman AS, HIT-COVID Collaboration (2020). HIT-COVID, a global database tracking public health interventions to COVID-19. Sci Data.

[46] Wright A (2016). REDCap: a tool for the electronic capture of research data. Journal of Electronic Resources in Medical Libraries.

